# Quantitative N-glycoproteomic analysis reveals glycosylation signatures of plasma immunoglobulin G in systemic sclerosis

**DOI:** 10.3389/fimmu.2025.1531191

**Published:** 2025-02-07

**Authors:** Lu Cheng, Yanhong Li, Yu Zhou, Yingying Ling, Tong Wu, Zongan Liang, Yinlan Wu, Chunyu Tan, Yi Liu, Yong Zhang

**Affiliations:** ^1^ Department of Pulmonary and Critical Care Medicine, State Key Laboratory of Respiratory Health and Multimorbidity, Institutes for Systems Genetics, West China Hospital, Sichuan University, Chengdu, China; ^2^ Department of Rheumatology and Immunology, Laboratory of Rheumatology and Immunology, West China Hospital, Sichuan University, Chengdu, China; ^3^ Department of Respiratory and Critical Care Medicine, Chengdu First People’s Hospital, Chengdu, China

**Keywords:** systemic sclerosis (SSc), N-glycosylation, glycopeptide, immunoglobulin g (IgG), glycoproteomics

## Abstract

Systemic sclerosis (SSc) is a perplexing autoimmune disorder, characterized by mysterious causes, high mortality rates, and a lack of effective treatments. The role of abnormal glycosylation in the onset of autoimmune diseases has been recognized for some time. Nonetheless, the intricate details of intact glycopeptides in SSc remain elusive owing to challenges in their detection. In this study, we characterized plasma immunoglobulin G (IgG) intact N-glycopeptides from 30 SSc patients and 30 healthy controls (HCs) via our recently developed intact glycopeptide analysis method GlycoQuant. Through this approach, twelve differentially expressed intact N-glycopeptides were identified. The correlation of specific intact N-glycopeptides with the clinical features of SSc patients was analyzed. The results revealed a notable increase in the levels of 6 intact N-glycopeptides (IgG2-N3H3F1, IgG2-N3H4F1, IgG2-N4H4F1, IgG2-N4H5F1, IgG2-N5H4F1, and IgG2-N5H5F1) and a decrease in the levels of another set of 6 intact N-glycopeptides (IgG1-N4H3F1, IgG2-N3H6F1A1, IgG2-N4H4F1A1, IgG2-N5H3F1, IgG3-N4H3F1, and IgG3-N4H4F1). These changes in the levels of intact N-glycopeptides are associated with various aspects of SSc, including diffuse SSc (dSSc), interstitial lung disease (ILD), disease progression, cardiovascular involvement and C-reactive protein in the peripheral blood. In summary, this study offers a detailed overview of the intact N-glycopeptide profile in the peripheral blood of patients with SSc, providing valuable insights that could propel further research into SSc.

## Introduction

Systemic sclerosis (SSc) is a rare yet severe autoimmune disorder, marked notably by its progressive fibrosis, which primarily manifests as thickening, hardening, and fibrosis of the skin (1). Beyond its dermatological impact, SSc extends its reach to visceral organs including the lungs, heart, kidneys, and digestive system, among others. SSc is underpinned by three fundamental pathological features: vascular lesions, immune system disorders, and fibrosis, which present a complex interplay of mechanisms, with the most critical aspects still shrouded in mystery (1). Despite the introduction of new medications aimed at managing SSc in recent years, their efficacy remains modest. Consequently, SSc continues to be the predominant cause of mortality among all autoimmune diseases (2). Disease-specific autoantibodies, including anti-centromere antibodies (ACA), anti-topoisomerase I/Scl-70 antibodies, and anti-RNA polymerase (RNAP) I-III, are of great value in the diagnosis and prognosis of SSc (1).

Glycosylation, a critical post-translational modification of proteins, predominantly occurs via N-glycosylation, the most prevalent form of this process (3, 4). This modification is not only widespread but also crucial in the function of many antibodies that play significant roles in autoimmune diseases (5–7). Immunoglobulin G (IgG) molecules are critical glycoprotein antibodies generated by B cells upon antigen exposure that specifically target and bind to the corresponding antigen. They are categorized into four subtypes: IgG1, IgG2, IgG3, and IgG4 (8). IgG recognizes and specifically binds antigens through its fragment antigen-binding (Fab) region, while fragment crystallizable (Fc) region mediates various antibody-dependent functions by binding to Fcγ receptors on different effector cells (8, 9). The primary factor influencing IgG function is the process of glycosylation (10). Both IgG Fab region and Fc region have N-glycosites, which have important effects on the stability, activity and half-life of IgG (10). The precise role that Fab glycosylation plays in immunity remains somewhat limited. In recent years, several studies have revealed that Fab glycosylation can regulate the antigen-binding affinity of IgG, thus exerting an impact on immunity. However, when compared to Fc glycosylation, it doesn’t show a strong correlation with disease progression (10). On the other hand, Fc glycosylation is more widely acknowledged. The N-glycosylation within the Fc region of IgG influences its binding to Fcγ receptors, which in turn regulates a host of downstream functions, such as cytotoxicity (ADCC), cellular phagocytosis (ADCP), neutrophil phagocytosis (ADNP), and complement deposition (ADCD) (11).

As a matter of fact, an increasing number of studies have reported on the changes in IgG N-glycosylation within autoimmune diseases like rheumatoid arthritis (RA) and systemic lupus erythematosus (SLE). RA patients display specific glycosylation patterns, which involve low levels of IgG galactosylation, low sialylation, and high fucosylation. What’s more, these abnormal glycosylation patterns shift along with the progression or remission of RA (12). Even more intriguingly, these specific glycosylation patterns start to change prior to the emergence of clinical symptoms (13). Decreases in galactosylation, sialylation, and core fucosylation have also been detected in the serum of SLE patients, and these changes are associated with organ involvement (14). The specific alterations in glycosylation patterns in ANCA-associated vasculitis might serve as a handy tool for predicting disease recurrence (15). All of this implies that changes in glycosylation could potentially drive the progression of inflammation, and the detection and analysis of IgG glycosylation patterns are crucial for monitoring disease activity. Nevertheless, information regarding IgG glycosylation in SSc is still rather scarce. One study that looked into the N-glycosylation of IgG in a cohort of 298 SSc patients demonstrated a significant decline in the levels of IgG galactosylation (16). Another study quantitatively analyzed the bleomycin-induced glycosylation in the Fc region of serum IgG in a mouse model of SSc and discovered that each IgG subclass exhibited distinct and unique glycosylation modifications (17). Despite these advances, the glycoproteomic landscape among SSc patients remains largely unexplored, especially when it comes to the quantitative analysis of intact N-glycopeptides in plasma IgG. These findings suggest that N-glycosylation acts as a crucial regulatory mechanism within the Fc region of IgG, toggling between the promotion of anti-inflammatory and pro-inflammatory responses. Understanding these glycosylation patterns of IgG not only provides insights into the complex interactions within the immune system but also creates opportunities for more accurate disease diagnosis and the development of targeted treatments. This exploration of the role of IgG glycosylation in health and disease underscores the potential for significant advancements in medical science.

The intricate nature of glycosylation positions it as a potential biomarker and target for intervention in the context of disease diagnosis and treatment. However, this characteristic also increases the complexity of glycoproteomic research. Recently, novel electron-transfer/higher-energy collisional dissociation (EThcD) and stepped collision energy/higher-energy collisional dissociation (sceHCD) mass spectrometry (EThcD-sceHCD-MS/MS) have become valuable methods for clinical glycoproteomics (18–20). This technique stands out for its depth and precision in identifying intact glycopeptides, marking significant strides in glycosylation research related to various diseases (21, 22). We also have established an integrated pipeline for site-specific quantification of N-glycosylation (termed GlycoQuant) (22). In this study, we characterized intact N-glycopeptides of plasma IgG from SSc patients and HCs via the GlycoQuant method, setting the stage for a deeper exploration into the glycoproteomic intricacies of SSc.

## Materials and methods

### Patient selection and biospecimen collection

A cohort of thirty patients diagnosed with SSc between 2021 and 2022 at the Department of Rheumatology, West China Hospital, Sichuan University, was carefully selected and enrolled in this study. All patients met the 2013 American College of Rheumatology/European League Against Rheumatism (ACR/EULAR) criteria, and 30 age- and gender-matched HCs from a cohort of 54 healthy individuals were included for comparison in this study (23). The detailed criteria are presented as follows: If the skin thickening of the fingers extends proximal to the metacarpophalangeal joints, it suffices for the patient to be classified as having SSc. In the absence of this feature, seven additive items come into play, each carrying different weights. These items include skin thickening of the fingers, fingertip lesions, telangiectasia, abnormal nailfold capillaries, interstitial lung disease or pulmonary arterial hypertension, Raynaud’s phenomenon, and SSc-related autoantibodies. Patients with severe infections, tumors or other autoimmune diseases were excluded. All the subjects signed an informed consent form prior to specimen collection. This study was approved by the ethical committee of West China Hospital of Sichuan University (No. 90 in 2022) and conducted in strict accordance with the Declaration of Helsinki. Blood samples are collected on the day following admission, in conjunction with other blood samples that are routinely collected during the patient’s hospitalization, such as samples for autoantibody profiles. Following the acquisition of informed consent, a five-milliliter peripheral blood sample was collected in an ethylenediaminetetraacetic acid disodium salt (EDTA) tube. The samples were then centrifuged at 1,200 g for 10 minutes at 4°C to separate the plasma, which was subsequently stored at -80°C until further analysis.

### Clinical and laboratory data collection

The clinical data were gathered directly from the patients’ medical records, and their plasma samples were collected. These data included demographic characteristics, disease history, complications and comorbidities, routine blood parameters, autoantibody profiles and inflammatory markers.

### Isolation, purification and digestion

The process of isolating, purifying, and digesting IgGs involves a series of meticulous steps (24). Briefly, a mixture of 40 μL of immobilized protein A/G agarose and 200 μL of binding buffer was combined with 20 μL of plasma. This mixture was then incubated at 4°C for two hours on a rotator to ensure thorough binding. Following this incubation period, any proteins that did not bind were washed away with 500 μL of binding buffer. To release the IgGs, 50 μL of elution buffer containing 0.1 M formic acid was added. This mixture was then incubated for five minutes at 25°C on a rotator. The eluate was neutralized by adding approximately 50 μL of neutralization buffer until it reached a physiological pH. The final step in the process was quantifying the IgG concentration. This was achieved via a bicinchoninic acid (BCA) protein assay (Thermo Fisher Scientific, USA) at 562 nm.

Purified IgGs (20 μg) were denatured by heating for 10 min at 95°C. Reduction was performed with 20 mM dithiothreitol (DTT) for 30 min at 56°C, and alkylation was performed with 50 mM iodoacetamide (IAA) for 30 min at 25°C in the dark. The mixture was then transferred into a 30 kDa ultrafiltration tube (Millipore, Bedford, MA, USA) for subsequent substitution with 50 mM ammonium bicarbonate (NH_4_HCO_3_) buffer (pH 8.5). Finally, 0.5 μg of sequencing grade trypsin (Promega, Madison, WI, USA) was added, and the mixture was incubated for two hours at 37°C. The tryptic IgG peptides were quantified via a colorimetric peptide assay (Thermo Fisher Scientific, USA) at 480 nm.

### LC-MS/MS analysis

Peptide analysis was conducted via an Orbitrap Fusion Lumos mass spectrometer (Thermo Fisher Scientific, USA) equipped with a nanoelectrospray ionization source and an EASY-nLC 1200 high-performance liquid chromatography system (Thermo Fisher Scientific, USA) as previously described (24).

More precisely, the peptides were dissolved in buffer A (buffer A, 0.1% FA in water; buffer B, 0.1% FA in 80% ACN) and separated on a 20-centimeter column (ReproSil-Pur C18-AQ, 1.9 μm, 75 μm inner diameter; Dr Maisch) with a 30-min gradient (0-2 min, 5-12% B; 2-7 min, 12-22% B; 7-21 min, 22-32% B; 21-22 min, 32-90% B; 22-30 min, 90% B) at a flow rate of 350 nL/min. The separated samples were subjected to EThcD-sceHCD-MS/MS. The parameter settings were outlined following previously established methods (22).

### Data analysis and bioinformatics

The process of identifying intact N-glycopeptides was conducted via Byonic software (version 3.10, Protein Metrics, Inc.), which specifically targets the human IgG database. The search parameters were finely tuned, with a mass tolerance set to ±6 parts per million (ppm) for precursor ions and ±20 ppm for fragment ions. The analysis allowed for up to two missed cleavage sites in trypsin digestion. Carbamidomethylation (C) was set as a fixed modification, whereas oxidation (M) and acetylation (protein N-term) were set as variable modifications. In addition, 182 human N-glycans were specified as N-glycan modifications. Protein groups were filtered to a 1% false discovery rate, and a confident intact N-glycopeptide required a score of no less than 200 and at least six amino acids. Furthermore, all glycopeptide-spectrum matches (GPSMs) were manually examined. The process of quantifying intact N-glycopeptides was performed via PANDA software (v.1.2.5). The label-free quantification mode was selected. This involved importing both MS raw files and intact N-glycopeptide identification files. Default values were used for all other parameters.

We performed data analysis via R programming software. Initially, we employed median normalization to mitigate technical variances across the samples. We subsequently pinpointed differentially expressed intact N-glycopeptides by leveraging the moderated t test available in the limma package. Additionally, we constructed principal component analysis (PCA) plots, heatmaps, and boxplots via R programming to visualize our findings. For supplementary data analysis, we used SigmaPlot 12.5 and GraphPad Prism 9.5 software. The results are depicted as either the mean ± standard deviation (M ± SD) or the median (Q_25_, Q_75_). A t test was conducted for comparisons between two groups. The analysis of correlations was performed via either the Pearson or Spearman correlation coefficient, which is based on the data type. We considered *p* values less than 0.05 to indicate statistical significance.

## Results

### Experimental design

**Figure 1:**
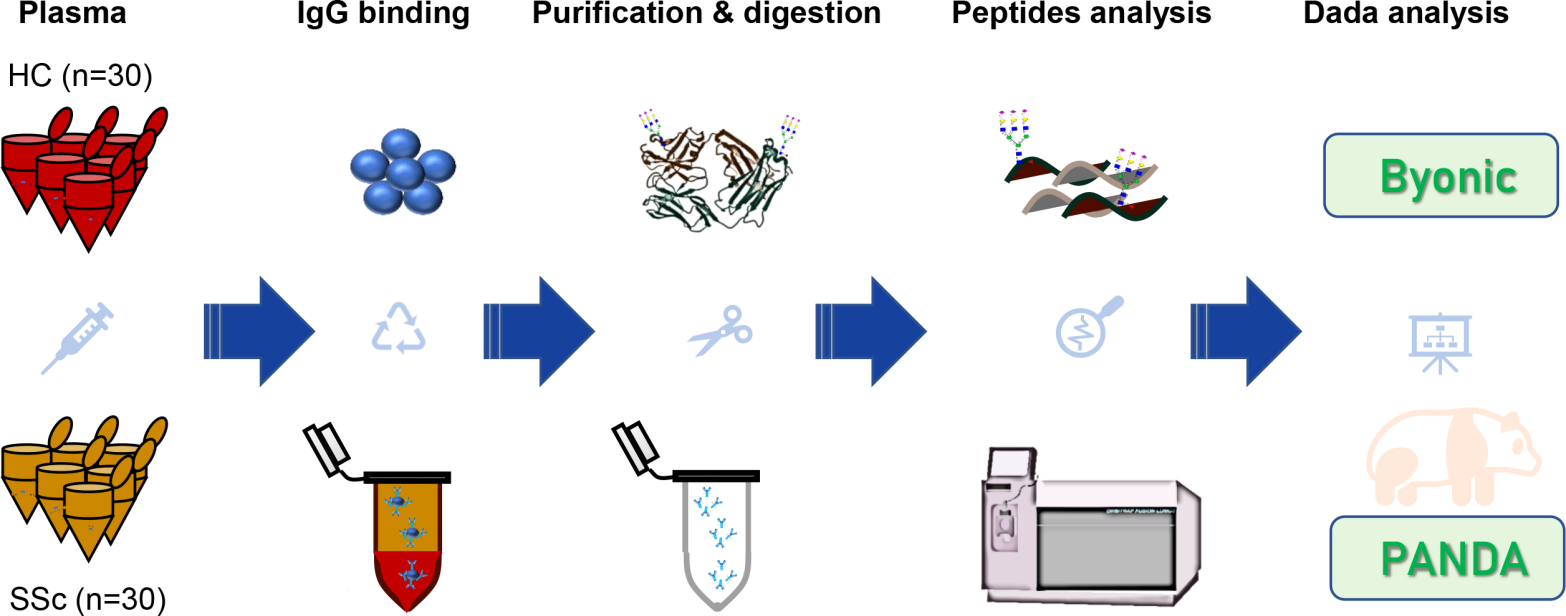
Schematic representation of the workflow for IgG intact N-glycopeptide analysis.

In recent years, the characteristics of plasma IgG glycosylation in many diseases have been explored via liquid chromatography tandem mass spectrometry (LC-MS/MS), lectin affinity chromatography, autoantigen-specific immunoassays and other methods (25–27). However, the quantitative characteristics of plasma IgG intact N-glycopeptides from SSc patients have not been reported. To investigate the expression changes and clinical relevance of IgG glycosylation in SSc, we designed the following experimental workflow (**Figure 1**). In this study, plasma IgG molecules from 30 SSc patients and 30 HCs were isolated, purified and digested. The obtained peptides and intact N-glycopeptides were analyzed via EThcD-sceHCD-MS/MS (18, 24, 28). The identification and quantification of intact N-glycopeptides were performed via Byonic and PANDA software, respectively. Ultimately, we aimed to identify SSc-associated differentially expressed IgG glycosylations and their relationships with clinical features.

### Baseline demographics and clinical characteristics

The baseline demographics and clinical characteristics of the individuals included in this study are outlined in **Supplementary Table S1**. A total of 30 SSc patients and 30 HCs were meticulously recruited and enrolled in this study. The average ages in the HC and SSc groups were 50.63 ± 6.87 and 50.07 ± 1.71 years, respectively. The median disease duration among SSc patients was 4 years, with a preponderance of patients exhibiting diffuse cutaneous SSc (80%), interstitial lung disease (ILD, 80%), and a positive status for anti-Scl-70 autoantibodies (83.33%). The average dosage of glucocorticoids administered was 22.83 mg, and more than half of the patients received cyclophosphamide therapy. The features of the patients with SSc included in this study are consistent with the general characteristics of SSc disease (1). The general characteristics are important for the diagnosis and management of SSc.

### N-glycosylation characterization of plasma IgG in patients with SSc

**Figure 2:**
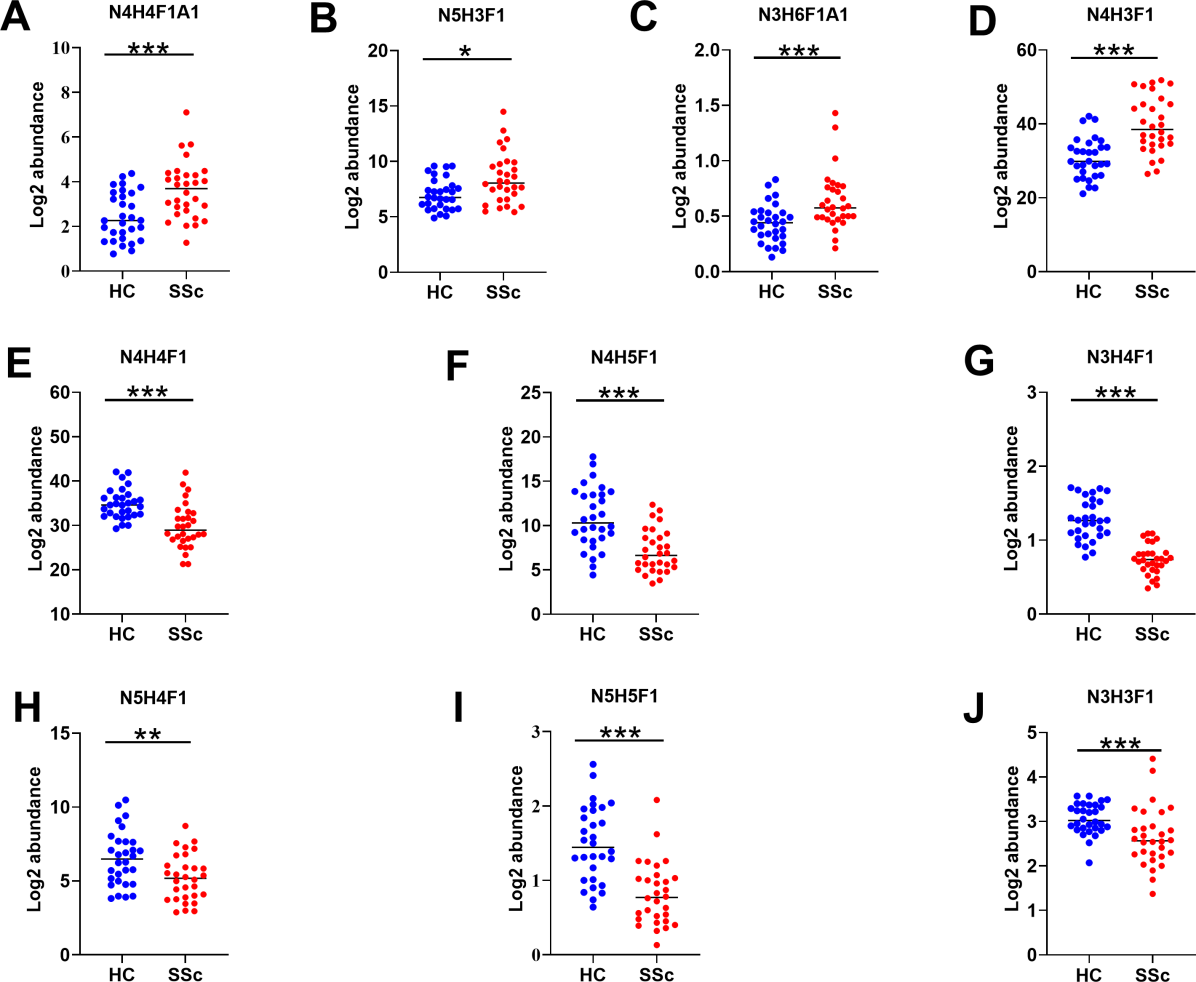
Characteristics of IgG N-glycans in patients with SSc. Panels **(A–J)** illustrate the key N-glycosylation traits that significantly differ between the HC and SSc groups. H, hexose; N, N-acetylhexosamine; F, fucose; A, N-acetylneuraminic acid; Statistical differences between the SSc and HC groups are as follows: *p <0.05, **p<0.005, ***p<0.001.

**Figure 3:**
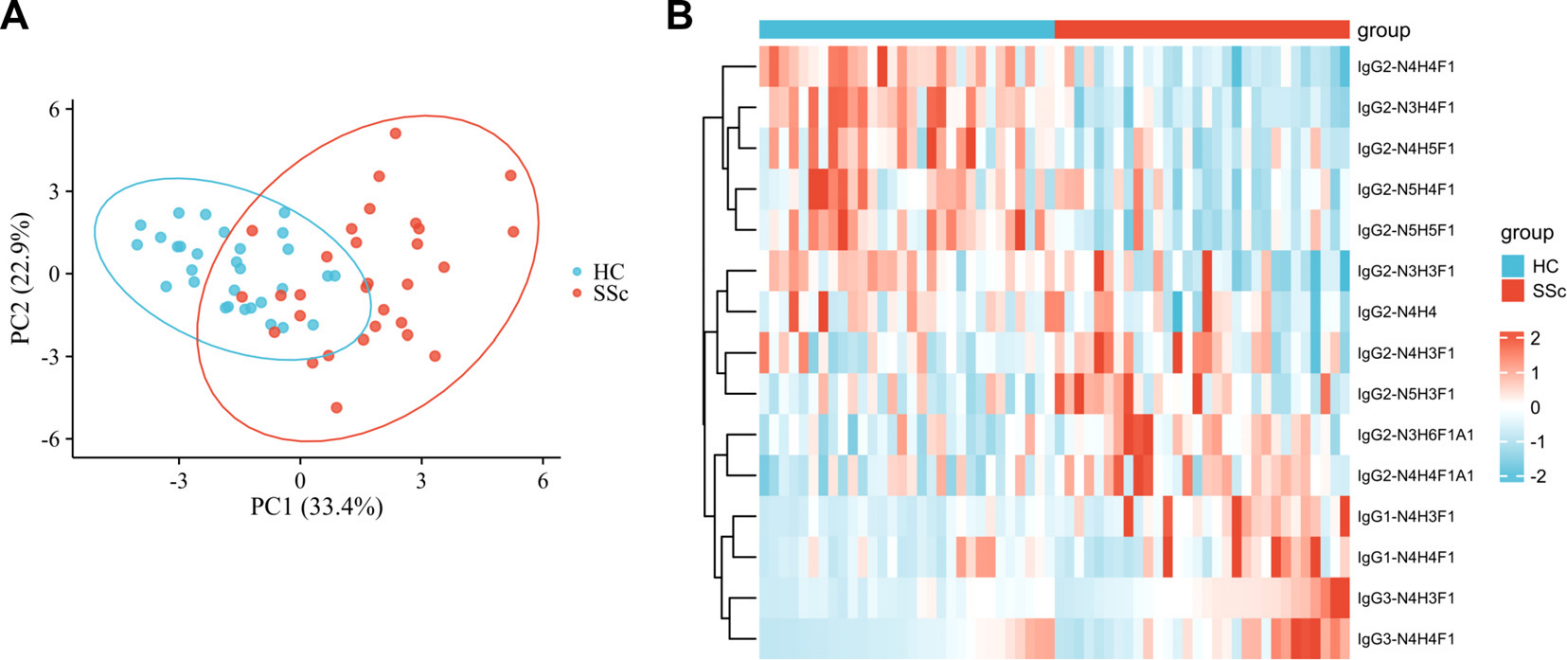
IgG N glycopeptides in SSc patients are different from those in HC patients. **(A)** The PCA plot demonstrates that these intact N-glycopeptides effectively differentiate between HC individuals and SSc patients. **(B)** The variations in intact N-glycopeptides of IgG between the HC and SSc groups were revealed. The subjects with the lowest quantitative values are represented in blue, whereas those with the highest quantitative values are depicted in red. The heatmap displays the relative minimum (blue, -2) and maximum (red, 2) values for each row.

**Figure 4:**
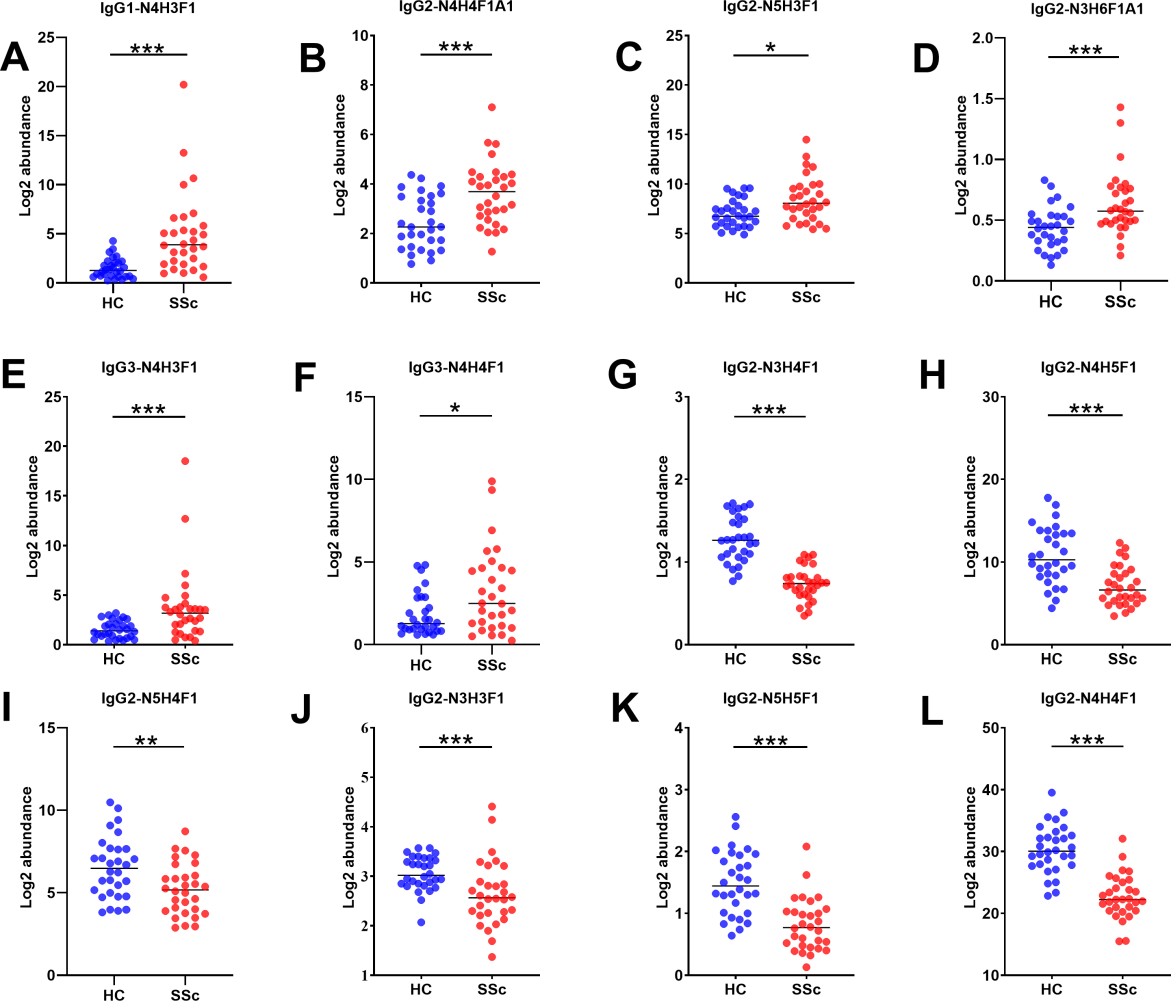
Characteristics of IgG subclasses of intact N-glycopeptides in patients with SSc. Panels **(A–L)** highlight the key intact N-glycopeptide traits that significantly differ between the HC and SSc groups. Statistical differences between the SSc and HC groups are as follows: *p <0.05, **p<0.005, ***p<0.001.

We performed a qualitative and quantitative analysis of IgG intact N-glycopeptides from the SSc and HC groups. A total of 11 highly reliable and reproducible N-glycans were quantified from both groups (**Supplementary Table S2**). To simplify the N-glycan structure, we used abbreviations (e.g., N3H3F1), where the labeling corresponds to the N-glycan composition represented in hexose (H), N-acetylhexosamine (N), fucose (F), and N-acetylneuraminic acid (A). The results of the quantification of the IgG N-glycans in the two groups were compared. As shown in **Figure 2**, we detected 4 increased (**Figures 2A-D**) and 6 decreased (**Figures 2E-J**) IgG N-glycans in patients with SSc compared with HCs. Importantly, the sialylation of IgG was significantly elevated (*p*<0.001). Furthermore, 15 highly reliable and reproducible intact N-glycopeptides of IgG subclasses (IgG1, IgG2 and IgG3) were quantified from both groups (**Supplementary Table S3**). Principal component analysis (PCA) demonstrated that these IgG intact N-glycopeptides effectively distinguished between the HC and SSc groups (**Figure 3A**). Heatmap analysis revealed differences in intact N-glycopeptides between SSc and HC groups (**Figure 3B**). Further analysis of the intact N-glycopeptides of the IgG subclasses revealed that all 6 down-regulated intact N-glycopeptides were from IgG2, namely, IgG2-N3H3F1, IgG2-N3H4F1, IgG2-N4H4F1, IgG2-N4H5F1, IgG2-N5H4F1, and IgG2-N5H5F1 (**Figures 4A–F**). In addition, 6 up-regulated intact N-glycopeptides included IgG1-N4H3F1, IgG2-N3H6F1A1, IgG2-N4H4F1A1, IgG2-N5H3F1, IgG3-N4H3F1, IgG3-N4H4F1 (**Figures 4G–L**). Besides, the levels of IgG1-N4H4F1, IgG2-N4H3F1 and IgG2-N4H4 did not differ significantly between the two groups (**Supplementary Figure S1**).

### Correlation between IgG glycosylation and the clinical features of SSc patients

**Figure 5:**
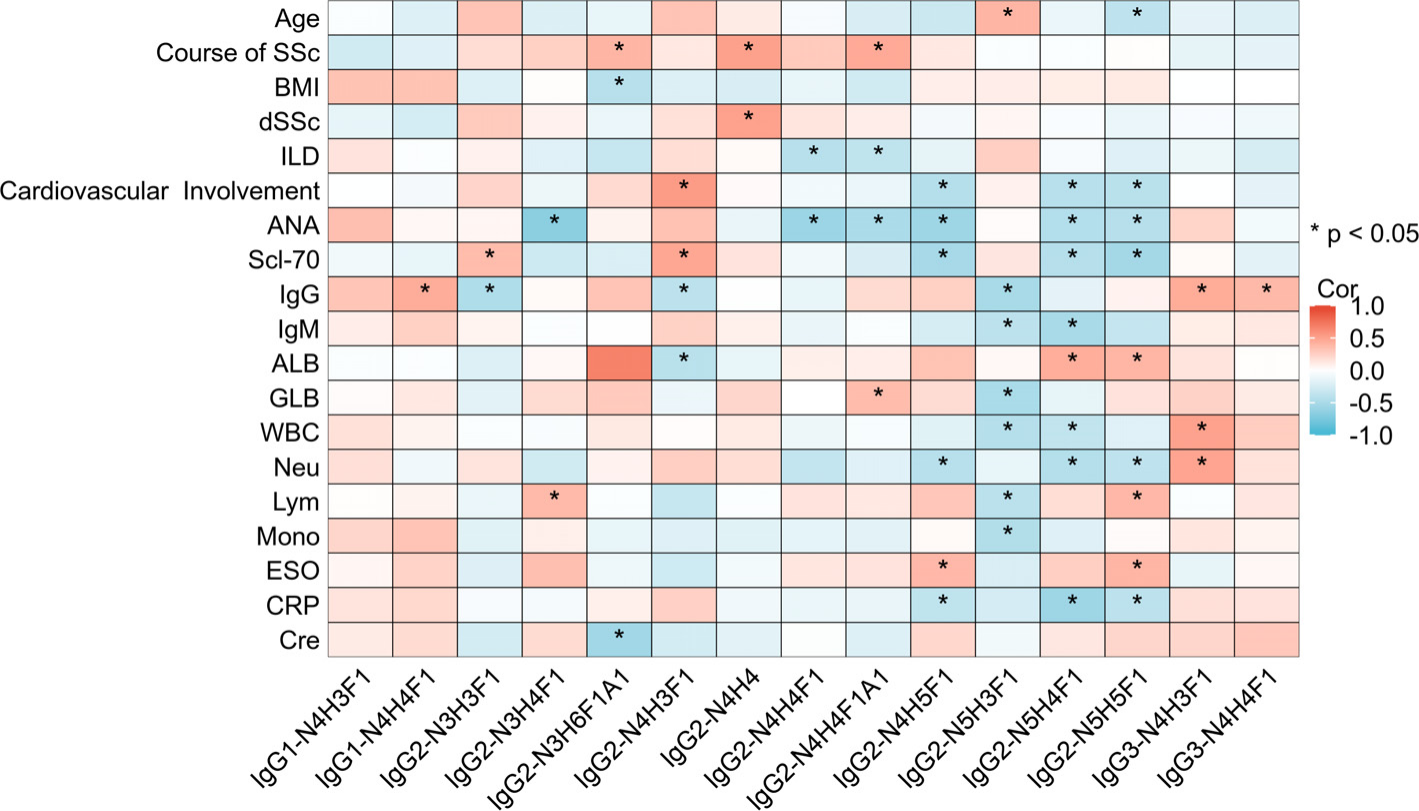
Heatmap of the correlation between intact N-glycopeptides and the clinical features of SSc patients. dSSc, diffuse SSc; ANA, antinuclear antibody; IgG, immunoglobulin G; IgM, immunoglobulin M; ALB, albumin; GLB, globulin; WBC, white blood cell; Neu, neutrophils; Lym, lymphocytes; Mono, monocytes; ESO, erythrocyte sedimentation rate; CRP, C-reactive protein; Cre, creatinine. *p <0.05.

To explore the significance of IgG glycosylation in relation to the clinical aspects of SSc, we performed a correlation analysis between the clinical features of SSc and intact N-glycopeptides (**Figure 5**; **Supplementary Table S4**). Specifically, the age of SSc patients was positively correlated with IgG2-N5H3F1 (r=0.403, *p*=0.027) and negatively correlated with IgG2-N5H5F1 (r=-0.385, *p*=0.035). Additionally, the duration of SSc was found be positively correlated with IgG2-N3H6F1A1, IgG2-N4H4 and IgG2-N4H4F1A1 (r=0.404, 0.512 and 0.464, respectively; all *p*<0.01). IgG2-N4H4 was positively correlated with diffuse SSc (r=0.51, *p*=0.004). IgG2-N4H4F1 and IgG2-N4H4F1A1 were negatively correlated with interstitial lung disease (r=-0.423 and -0.378, respectively, *p*<0.05). Cardiovascular involvement was positively correlated with IgG2-N4H3F1 (r=0.543, *p*=0.002) and negatively correlated with IgG2-N4H5F1 (r=-0.439, *p*=0.015). The antinuclear antibody (ANA) titer was negatively correlated with the IgG2-N3H4F1, IgG2-N4H4F1, IgG2-N4H4F1A1, IgG2-N4H5F1, IgG2-N5H4F1 and IgG2-N5H5F1(r=-0.669, 0.581, -0.5, 0.561, -0.45 and -0.436, respectively; all *p*<0.05). The anti-Scl-70 antibody was positively correlated with IgG2-N3H3F1 and IgG2-N4H3F1 (r=0.366 and 0.482, respectively; *p*<0.05) and negatively correlated with IgG2-N4H5F1, IgG2-N5H4F1 and IgG2-N5H5F1 (r=-0.519, -0.443 and -0.538, respectively, all *p*<0.05). The peripheral blood white blood cell (WBC) count was negatively correlated with IgG2-N5H3F1 and IgG2-N5H4F1 (r=-0.439 and -0.374, respectively; *p*<0.05), and positively correlated with IgG3-N4H3F1 (r=0.507, *p*<0.05). The peripheral blood neutrophil count was negatively correlated with IgG2-N4H5F1, IgG2-N5H4F1 and IgG2-N5H5F1 (r=-0.424, -0.446 and -0.384, respectively; all *p*<0.05) and positively correlated with IgG3-N4H3F1 (r=0.497, *p*=0.005). The peripheral blood lymphocytes were positively correlated with the IgG2-N3H4F1 and IgG2-N5H5F1 (r=0.379 and 0.39, respectively; *p*<0.01), and negatively correlated with IgG2-N5H3F1 (r=-0.414, *p*=0.033). The peripheral blood monocyte count was negatively correlated with IgG2-N5H3F1 (r=-0.46, *p*=0.01). The erythrocyte sedimentation rate (ESR) was positively correlated with IgG2-N4H5F1 and IgG2-N5H5F1 (r=0.385 and 0.405, respectively; *p*<0.05). C-reactive protein (CRP) was negatively correlated with IgG2-N4H5F1, IgG2-N5H4F1 and IgG2-N5H5F1 (r=-0.376, -0.569 and -0.401, respectively; all *p*<0.05).

## Discussion

We performed qualitative and quantitative analysis of the IgG subclasses of intact N-glycopeptides from 30 patients with SSc and 30 HCs via the GlycoQuant method (**Figure 1**). Twelve abnormally expressed IgG intact N-glycopeptides (including 6 up-regulated and 6 down-up-regulated ones) were found in SSc patients. These intact N-glycopeptides were also found to be associated with SSc clinical subtypes, specific organ manifestations and inflammation levels. These results suggest that human plasma IgG N-glycosylation may be involved in the progression of SSc.

The EThcD-sceHCD technique represents a cutting-edge method for the analysis of intact N-glycopeptides. Its effectiveness in analyzing intricate clinical samples has been validated, offering key insights for distinguishing between various chronic kidney diseases (24). Herein, we are the first to use this technology to quantify IgG intact N-glycopeptides from SSc patients. Our results reveal that SSc patients have a unique pattern of IgG N glycosylation. IgG sialylation is significantly up-regulated in patients with SSc (**Figure 2**). IgG sialylation plays an important role in tumor immune escape by inhibiting T-cell activity (29). Patients with SSc are known to have a significantly greater incidence of tumors, with lung cancer being the most prevalent (30). Interestingly, chronic obstructive pulmonary disease (COPD) is recognized as a risk factor for lung cancer, and patterns of IgG N-glycosylation are consistently linked to increased levels of IgG sialylation in lung cancer patients (31, 32). These findings indicates that abnormal IgG glycosylation may be involved in the susceptibility of SSc patients to lung cancer. Furthermore, we conducted correlation analysis between the identified intact N-glycopeptides and the clinical features of SSc patients (**Figure 5**). We discovered a negative correlation between IgG2-N4H4F1A1 and ILD in SSc patients. Fibrosis is a key characteristic of SSc, and ILD is the leading cause of death in patients with SSc (1). The early diagnosis and treatment of ILD is of great significance for the prognosis of SSc. Encouragingly, IgG N-glycosylation can be a promising biomarker. However, data on IgG glycosylation in SSc are limited. An analysis of IgG N-glycosylation in 298 patients with SSc revealed a significant decrease in IgG galactosylation levels, although other glycans were not analyzed (16). In addition, decreased levels of IgG sialic acid and galactosylation were observed in bleomycin-induced mouse models of SSc (17). It’s worth noting that alterations in IgG sialylation within autoimmune diseases have been thoroughly documented. The sialylation levels drop markedly in active autoimmune diseases and even prior to disease relapses (6). These findings imply that IgG sialylation holds great significance for both disease diagnosis and predicting the risk of recurrence. Furthermore, intervening in autoantibody sialylation might represent a potential avenue for disease treatment. At the same time, we should be aware that 80% of our cohort suffered from ILD, and all of them were treated with immunosuppressants. Hence, the characteristics of glycopeptides in SSc need to be interpreted with caution. Given the impact of factors like smoking, body mass index (BMI), etc. on the results of glycosylation, future studies must take into account and balance the influence of these factors (33, 34). Additionally, it’s essential to consider the possible variations in the glycopeptide profile at different stages of the disease (35). The establishment of early or preclinical SSc cohorts would be beneficial for the discovery of biomarkers that can facilitate the early identification of SSc.

Additionally, we found that IgG2-N4H3F1 and IgG2-N4H5F1 are associated with cardiovascular involvement. Specific anti-glycosylation antibodies are present in the plasma of SSc patients and are associated with pulmonary hypertension (36). Previous studies have reported a connection between IgG N-glycosylation and the risk of cardiovascular events (37). These findings underscore that the expression of intact N-glycopeptides changes as the disease progresses. These specifically expressed glycopeptides may be biomarkers for accurately identifying organ-specific damage. In addition, we also found that various types of N-glycopeptides in the peripheral blood of SSc patients are associated with different immune cells, such as lymphocytes, neutrophils, and monocytes. The N-glycosylation of IgG influences the activation of autoimmune cells, cell-cell interactions, and the production of autoantibodies (9). Although the current literature is limited, our findings suggest that distinct N-glycopeptides trigger diverse immune responses through specific and intricate mechanisms. Further investigations into how glycosylation affects the immune response could deepen our understanding of the pathogenesis of SSc. These correlation analysis results show that quantifying intact glycopeptides of some glycoproteins in clinical samples could be valuable for diagnosing, monitoring severity, and predicting the prognosis of SSc.

Additionally, the intact N-glycopeptides of IgG1 and IgG3 in patients with SSc were found to be up-regulated compared with those in HCs, whereas the intact N-glycopeptides of IgG2 were primarily down-regulated (**Figures 3**, **4**). There is significant variation in the biological activities of different IgG subclasses in autoimmune diseases (38, 39). The protein levels of IgG1 and IgG3 are significantly increased in SSc patients, whereas the level of IgG2 is decreased (40). This result is almost consistent with our findings of IgG intact N-glycopeptide levels.

Our study has several limitations. First, the sample size is small, making it challenging to perform detailed subgroup analyses of SSc to assess its clinical application value. Our cohort is characterized by the preponderance of SSc patients with ILD complications, which makes our findings need to be interpreted in this context. Second, the details of the N-glycan structure are not resolved. Moreover, given the crucial role of disease-specific autoantibodies play in autoimmune diseases, it is essential to further identify the changes in the glycosylation spectrum of SSC-specific autoantibodies. This will contribute to a better understand of how abnormal glycosylation give rise to the pathogenesis of the disease. Finally, the functions of these intact N-glycopeptides remain unclear, necessitating further basic research to investigate the molecular mechanisms behind different glycosylation patterns in diseases.

## Conclusion

In this study, intact N-glycopeptides of IgG subclasses from patients with SSc and HCs were characterized qualitatively and quantitatively via the GlycoQuant method, and 12 differentially expressed intact N-glycopeptides were found to be associated with specific clinical features and potentially involved in disease progression. These abnormally expressed intact N-glycopeptides may serve as biomarkers of SSc development.

## Data Availability

The mass spectrometry data have been deposited to the Proteome Xchange Consortium via the iProX partner repository with the dataset identifier PXD057509.
