# Supplementary material for: Quantitative N-glycoproteomic analysis reveals glycosylation signatures of plasma immunoglobulin G in systemic sclerosis
Source: Front Immunol. 2025 Feb 7;16:1531191. doi: 10.3389/fimmu.2025.1531191 (PMC11842340; doi:10.3389/fimmu.2025.1531191)

**Supplementary Information**

**Quantitative N-glycoproteomic analysis reveals** **glycosylation signatures of plasma** **immunoglobulin G in systemic sclerosis**

Lu Cheng^1 #^, Yanhong Li^2 #^, Yu Zhou^3 #^, Yingying Ling^1 #^, Tong Wu^2^, Zongan Liang^1^, Yinlan Wu^2^, Chunyu Tan^2^, Yi Liu^2^ *, Yong Zhang^1^ *

^1^Department of Pulmonary and Critical Care Medicine, State Key Laboratory of Respiratory Health and Multimorbidity, Institutes for Systems Genetics, West China Hospital, Sichuan University, Chengdu 610041, China.

^2^Department of Rheumatology and Immunology, Laboratory of Rheumatology and Immunology, West China Hospital, Sichuan University, Chengdu 610041, China.

^3^Department of Respiratory and Critical Care Medicine, Chengdu First People’s Hospital, Chengdu 610095, China.

^#^These authors contributed equally.

*Corresponding authors:

Yong Zhang ([nankai1989@foxmail.com/zhangyong0809@wchscu.cn](mailto:nankai1989@foxmail.com/zhangyong0809@wchscu.cn)), Yi Liu (yiliu8999@wchscu.cn).

**Table of Contents**

**Supplementary Figure S1.** Undifferentiated intact N-glycopeptides are expressed in both the HC and SSc groups. Panels A-C illustrate these intact N-glycopeptides that show no significant differences between the HC and SSc groups.

**Supplementary Table S1.** Baseline demographics and clinical characteristics of all individuals.

**Supplementary Table S2.** Quantitative result of IgG N-glycans in the two groups.

**Supplementary Table S3.** Quantitative result of IgG intact N-glycopeptides in the two groups.

**Supplementary Table S4.** Correlation between IgG glycosylation and clinical features of SSc.

**Supplementary Figure S1.** Undifferentiated intact N-glycopeptides were expressed in both HC and SSc groups. Panels A-C illustrate these intact N-glycopeptides that showed no significant differences between HC and SSc groups.


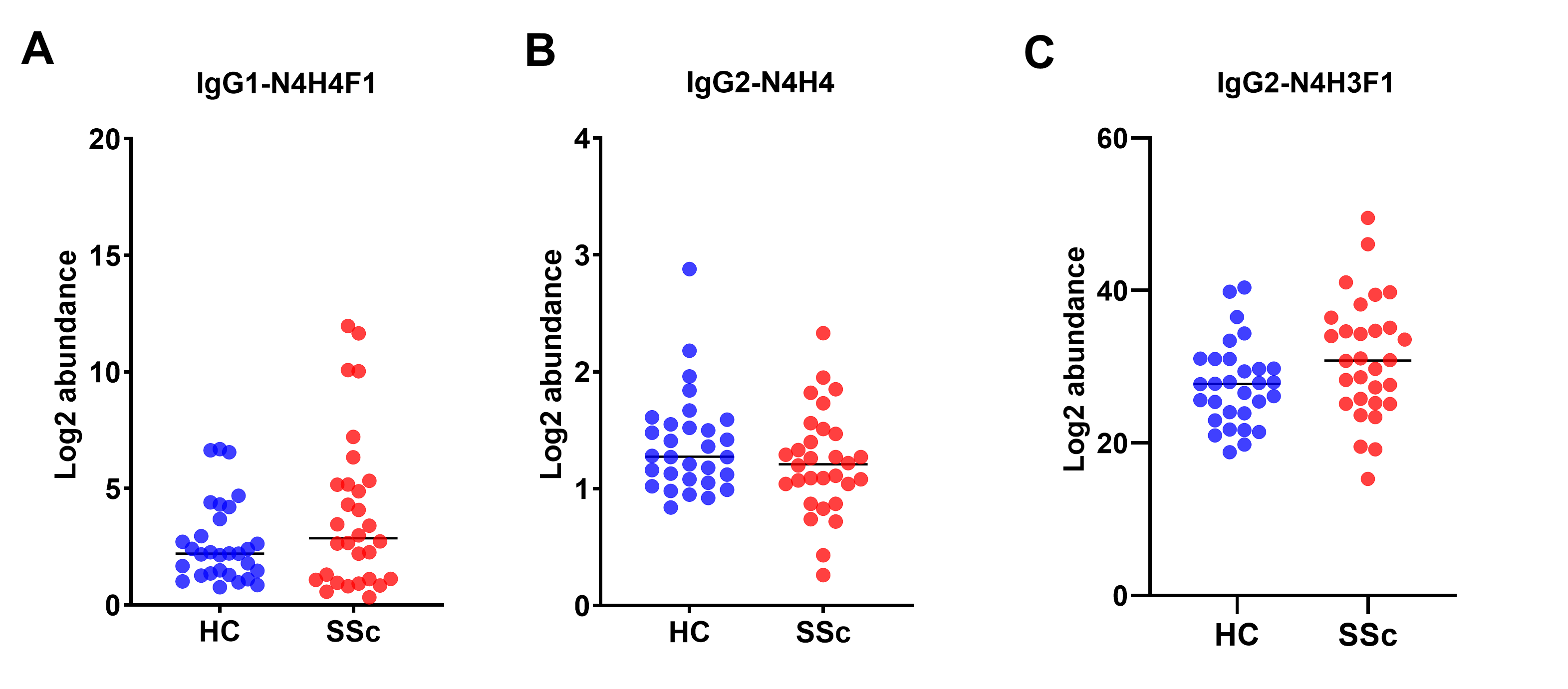

Supplement: Supplementary file 1 [file DataSheet1.doc]
